# Effect of Substrate Bias on the Microstructure and Properties of CrAlSiN Composite Coatings

**DOI:** 10.3390/nano16040278

**Published:** 2026-02-23

**Authors:** Huijin Song, Fan Zhao, Qiang Yan, Xin Zhao, Fan Lei, Ruijun Dong

**Affiliations:** 1 College of Mechanical Engineering, Chengdu University, Chengdu 610106, China; songhuijin@cdu.edu.cn (H.S.); zf1798966499@163.com (F.Z.); 17311680913@163.com (X.Z.);; 2 Chengdu Product Quality Inspection and Research Institute Co., Ltd., Chengdu 610200, China

**Keywords:** CrAlSiN nanocomposite, arc ion plating, substrate bias, mechanical properties

## Abstract

CrAlSiN nanocomposite coatings with different structures were prepared by arc ion plating. The influence of substrate bias on the composition, microstructure and properties of the coating was investigated. The nanocomposite CrAlSiN coatings all had a fcc-(Cr, Al)N phase, where Al atoms and some Si atoms were solid-dissolved in CrN phase and some Si existed in the form of amorphous phase in the coating. The coatings were preferentially grown along the (200) crystal plane. With the increase in substrate bias, the roughness of the coating gradually decreased. When the substrate bias gradually increased to 100 V, the small particles aggregated into large particles, producing more holes, so that the surface roughness of the coating increased. At the same time, with the increase in substrate bias, the hardness and adhesion of the coating first increased and then decreased. When the substrate bias voltage was 80 V, the coating had the largest hard H (31.30 GPa), elastic modulus E* (432.15 GPa), H/E* (0.0724), H^3^/E*^2^ (0.1642) and binding force of 109.26 N.

## 1. Introduction

With the development of science and technology, the process of cutting processing is becoming increasingly severe, and the requirements for tool coating are getting higher and higher. A single coating cannot meet the requirements of cutting processing, so the coating has developed from the original binary compound to today’s ternary-composite coating, multi-component nano-composite coating, high entropy alloy coating or nano-multilayer coating, such as CrAlN, AlSiN, TiSiN-WS_2_, TiC-TiN-A1_2_O_3_ composite coating and (Cr, Al)N, Ti, CrAl(Si)N and other multi-component composite coatings [1,2].

Si is doped in CrAlN coatings can further improve the mechanical properties and tribological properties of the coating [3,4,5,6,7]. In multilayer ZrN/CrN nanocomposite coatings, ZrN/CrN nanocomposite coatings with a 30-bilayer superlattice structure have resistance to plastic deformation and wear resistance [8]. AlCrN coating, AlTiN/Si_3_N_4_ coating, AlCrN/Si_3_N_4_ nanocomposite coating, and AlCrN/Si_3_N_4_ nanocomposite coating shows superior bonding performance, anti-high-temperature wear and anti-high-temperature oxidation [9]. Al in the coating is easy to react with O in the air to generate a dense Al_2_O_3_ film; doped Si is easy to react with N to generate Si_3_N_4_, which resists grain growth, and the fine grain strengthening effect is significant in the actual processing application [10,11,12,13]. A high-plasticity amorphous layer Si_3_N_4_ wrapped with high-hardness AlN, CrN, and TiN nanocrystal forms the nanocomposite structure, which can effectively improve the mechanical and tribological properties of the coating to increase the service life of the coated tool [14,15]. Chang, W., Cai, H., and Xue, Y. et al. reported that the H and E of CrAgCeN coatings were maximized (14.2 and 206.2 GPa, respectively) at 0.6 Pa deposition pressure [16]. Annealing-induced concomitant can improve the toughness and strength of the AlCrN thin films at grain boundaries, enhancing the cohesive energy of the grain boundaries and thereby altering the crack propagation pathway from inter- to transcrystalline [17]. Bias voltages of the substrate also play an important role in refining the grains, densifying the film, and inducing compressive residual stress. These mechanisms can improve hardness up to a point beyond the defect accumulation and amorphization occurring [18]. The increase in substrate bias can improve the kinetic energy of argon ions and deposited metal ions to effectively cleaning the surface and improving the adhesion of the film layer, resulting in a stronger sputtering effect when bombarding the substrate [6]. However, excessive bias voltage can lead to reverse sputtering, reduce deposition rate, and introduce defects, resulting in stress accumulation and decreased material plasticity.

In order to obtain better nano-hardness and elastic modulus of CrAlSiN composite coatings in cutting tools, arc ion plating was used; arc ion plating technology was used to prepare CrAlSiN composite coatings with different structures and nano-gradient CrAlSiN coatings with different Al and Si contents. The influence of the substrate bias voltage on the composition, microstructure and properties of the coating was studied.

## 2. Experiments and Methods

**Figure 1:**
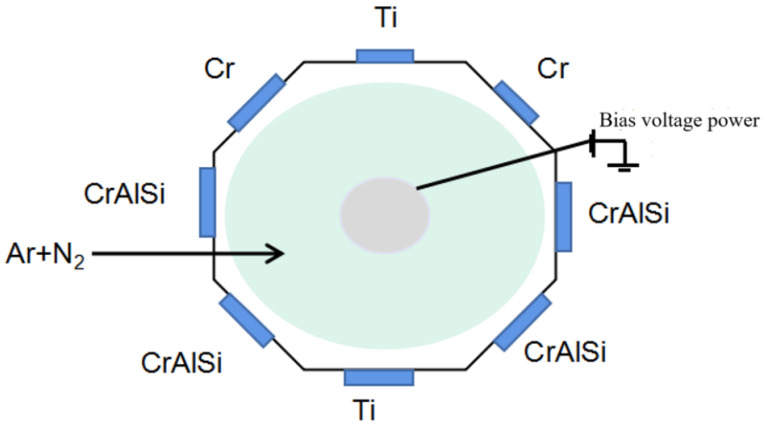
Target distribution.

CrAlSiN coatings were prepared by arc ion plating technology on the substrates of YT15 cemented carbide (10 mm × 10 mm × 3 mm) and a straight shank vertical milling cutter (Φ6 mm × 50 mm) with different deposition substrate bias voltages. The target material was Cr target (99.99%), CrAlSi target (6030:10), and Ti target (99.99%), where the Ti target was used for IET ion cleaning. Figure 1 shows the specific distribution of the target material.

The YT15 cemented carbide end mill was polished and ground in turn in the polishing machine, and then the samples and straight shank end mill were placed in the degreaser, using ultra-pure water and alcohol, respectively, for ultrasonic cleaning for 30 min. Then, the samples were fixed on a clamping plate and hung on the turning stand in the furnace after blowing dry with high-purity N_2_, keeping the samples vertical with a target base distance of 300 mm. The base vacuum in the coating chamber was evacuated to below 7.0 × 10^−3^ Pa, the temperature heated to 480 °C, and the rotation speed of the turntable was set to 50 r/min. Argon gas at 650 sccm was introduced, and the substrate bias was set to 500 V and 600 V for glow cleaning for 30 min. The argon flow rate was set to 150 sccm, the substrate bias was 40 V to 100 V, and the IET cleaning was carried out for 1 min, respectively, and then the Cr target was started, and the substrate surface was ion-bombarded and deposited, with the Cr layer as the transition layer by adjusting the bias, in order to enhance the bonding force between substrates and coatings. The bias was adjusted to 40 V, and nitrogen gas was introduced to make the furnace pressure 2.6 Pa; the Cr target was turned on for 20 min to prepare the CrN transition layer, and finally, the CrAlSi target was turned on together for 60 min, and the substrate biases were 40 V, 60 V, 80 V, 100 V to prepare the CrAlSiN layers. Table 1 shows the specific deposition parameters for the four coatings.

X-ray photoelectron spectroscopy (XPS) was analyzed by a multi-functional surface analysis system XSAM800 manufactured by British Kratos Company, Manchester, UK. The X-ray diffraction (XRD) measurement was performed with a RigukuDMAD-RCDX-1000 diffractometer using Cu Ka radiation by Rigaku Corporation of Japan, Tokyo, Japan. A Scanning Electron Microscope (SEM) was used with a Phenom XL produced by the FEI company, Hillsboro, OR, USA. The atomic force microscope (AFM) was obtained from Bruker MultiMode 8 from Ettlingen, Germany. The thickness of the coating was accurately measured by using the destructive testing method. We first ground the coating sample slices with a ball mill. After the coating was worn through, the thickness of the coating was accurately measured with a high-power microscope combined with the KaloMAX software NT II. The adhesion between coatings and substrates was tested by a scratch tester (ST200) manufactured by the German Fischer company, Achern-Fautenbach, Germany. The coating hardness test was tested by XSAM800 nanoindenter manufactured by British Kratos company.

## 3. Results and Discussion

### 3.1. Physical Analysis and Chemical Composition

**Figure 2:**
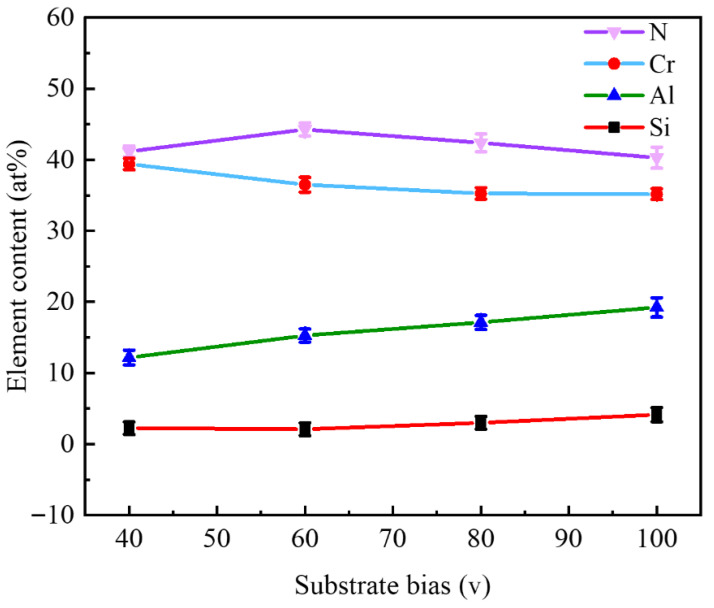
Chemical composition of CrAlSiN composite coatings with different substrate bias.

Figure 2 shows the chemical composition of four kinds of CrAlSiN nanocomposite coatings prepared by arc ion plating. It can be seen from Figure 2 that when the substrate bias voltage increased from 40 V to 100 V, the content of Al in the coating increased from 12.72 atm% to 19.05% for the increasing substrate bias voltage, and the mutual attraction between the charges made more Al deposit on the surface of the substrate. The content of Cr decreased slightly as the substrate bias voltage increased, as the decrease in Cr content caused the dissolution of Al atoms into Cr atoms. The low binding energy elemental Cr and limited availability of bonding partners of Al enhance the sputtering of Cr atoms. The content of Si fluctuated slightly but basically remained at about 2.8 at%. The content of N was maintained at about 4.0 at.%. In the thin film systems, higher N contents enable the formation of stable a-Si_3_N_4_ phase [19,20], thereby preventing Si resputtering.

**Figure 3:**
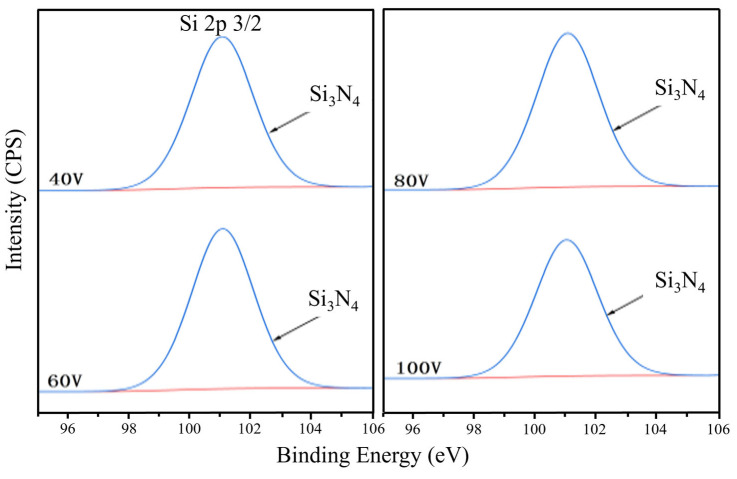
Si 2p3/2 XPS patterns of CrAlSiN composite coatings with different substrate bias.

**Figure 4:**
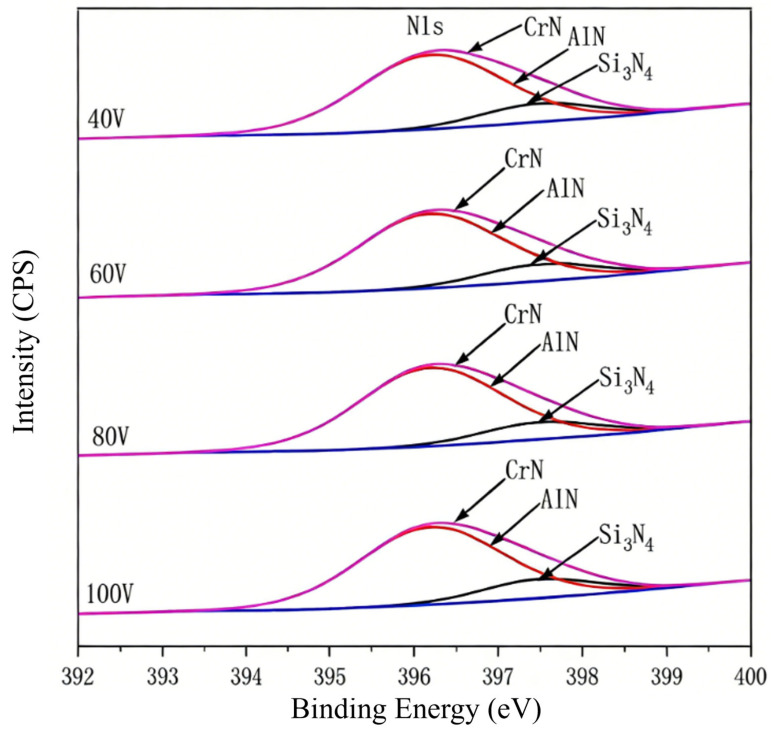
N 1s XPS patterns of CrAlSiN composite coatings with different substrate bias.

Figure 3 and Figure 4 give the Si 2p, N 1s spectrum results of CrAlSiN composite coating from X-ray photoelectron spectroscopy (XPS) analysis. It can be seen from Figure 3 that a unique 101.1 eV bond energy peak appears in the Si 2p spectrum of the four coatings, close to the bond of Si_3_N_4_. This indicates that there is amorphous Si_3_N_4_ in the four CrAlSiN coatings previously studied by researchers [21,22,23]. In the N 1s spectrum (Figure 4), a 397.5 eV bond energy peak also proves the existence of amorphous Si_3_N_4_. In addition, a bond energy peak of 396.2 eV also appeared in the Figure, close to the bond energy of AlN (396.2 eV) or CrN (396.4 eV).

**Figure 5:**
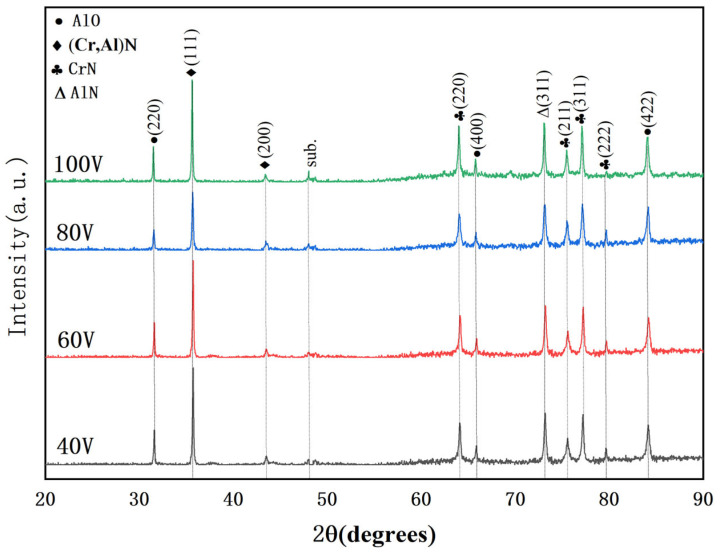
XRD spectra of CrAlSiN composite coatings with different substrate bias.

Figure 5 is the XRD diffractogram of four kinds of CrAlSiN composite coatings prepared under different substrate bias voltages. The four CrAlSiN coatings have detected the diffraction peaks of the (111) and (200) crystal surfaces of the fcc-(Cr, Al)N phase and other crystal surface diffraction peaks and AlO diffraction peaks. The peak width of the (111) crystal surface became wider first and then narrower, and the peak width of the (200) was opposite to the (111). During the coating process, in order to reduce stress and strain energy, the crystals inside the film formed along the direction of the lowest strain energy. In coating deposition, an increase in substrate bias voltage has a bidirectional effect on the diffraction peaks of the phase. On the one hand, when the bias voltage increases to a certain level, ionized particles bombard the substrate surface, causing lattice fracture and deformation within the deposited coating, leading to a significant increase in internal stress. The residual stress within the coating leads to the broadening or shifting of the diffraction peaks [24]. On the other hand, a high negative substrate bias voltage greatly increases the energy of the particles. According to the principle of energy conservation, a significant portion of this energy still reaches the substrate surface, causing a notable increase in substrate temperature, which not only allows the lattice structure within the coating that has been fractured and deformed to gradually recover but also promotes secondary nucleation and continued grain growth in the coating.

Films tend to grow following the crystal face with the lowest overall energy, which encompasses the surface energy and the stress energy. In view of the fact that the (200) face has a lower surface energy, the (111) crystal face exhibits the lowest stress energy [25]. Therefore, in a situation where the overall energy is by the surface energy, the film will choose to grow along the (200) crystal face, which has the lowest surface energy, for the growth of the crystal face [26,27]. According to the Scherrer calculation formula D = Kλ/βcosθ (where D represents the grain size, K is a constant (usually taken as 0.89), λ denotes the wavelength of X-rays, β signifies the Full Width at Half Maximum (FWHM) of the diffraction peak, and θ stands for the diffraction angle [28]), the grain sizes of four kinds of CrAlSiN composite coatings prepared under different substrate bias voltages were calculated. The grain sizes were 24.1 nm, 38.4 nm, 43.4 nm and 38.2 nm with substrate biases at 40 V, 60 V, 80 V and 100 V, respectively. So, the grain size was bigger with substrate bias at 80 V. The bigger grain sizes generally decrease the specific surface area and adsorption active sites of the material, thereby decreasing the adsorption performance. Grain size that is too small may lead to thermal instability, causing grain agglomeration to increase the surface roughness of the coatings.

### 3.2. Thickness of the Coatings

**Figure 6:**
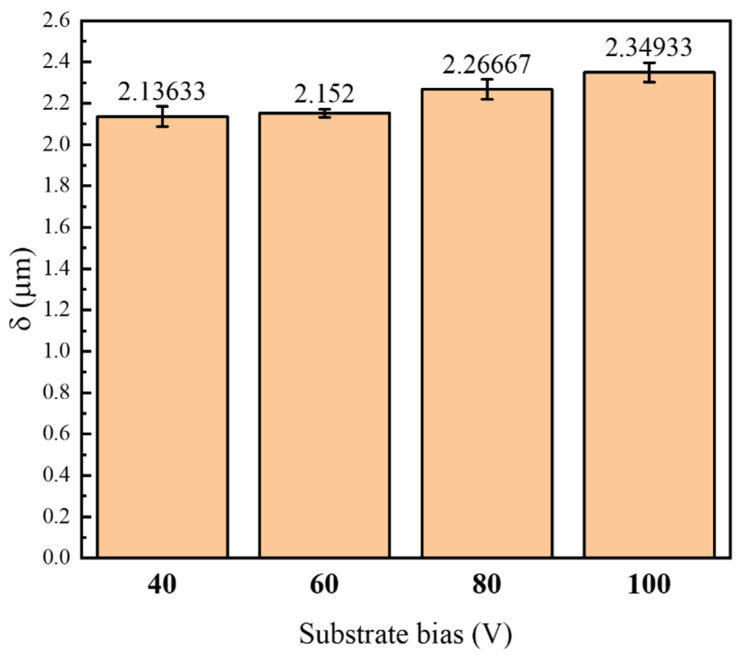
Thickness of CrAlSiN composite coating with different substrate bias voltages after ball grinding.

Figure 6 gives the thickness of CrAlSiN composite coating prepared at different substrate bias and then ball milled. It can be seen that with the bias increasing from 40 V to 100 V, the thickness of CrAlSiN coating increased from 2.116 μm to 2.52 μm with the same deposition time. Therefore, the increase in the thickness of the coating is approximately linearly related to the substrate bias. This is because the kinetic energy of ions is enhanced with larger bias voltage to move to the substrate with higher velocity. So, more ions would reach the substrate within a certain time and accelerate the charged particles and enhance their energy level, which benefits the generation with a more compact overlay. When the thickness is bigger, the hardness may increase, but the modulus of elasticity will decrease. A suitable thickness is very important. The thickness of CrAlSiN composite coating with different substrate biases was according to Qixiang Fan’s research [6].

### 3.3. Coating Surface Morphology

**Figure 7:**
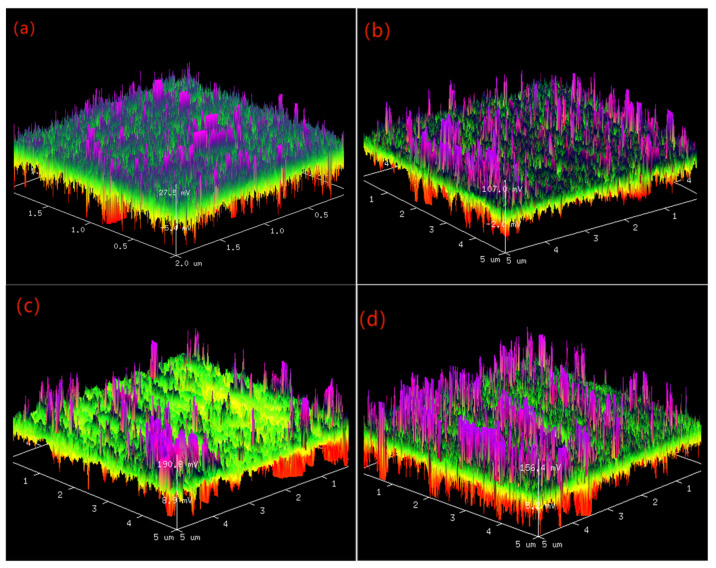
Three-dimensional surface topography of CrAlSiN composite coating with different substrate biases: (**a**) 40 V (**b**) 60 V (**c**) 80 V (**d**) 100 V.

Figure 7 shows the three-dimensional (3D) surface morphology of CrAlSiN coatings prepared under different substrate biases. When the substrate bias increased 40 V to 100 V, the average roughness (Ra) of the coating surface was 43.6 nm, 58.7 nm 37.8 nm, and 89.8 nm, respectively. The root mean square roughness (Rq) was 51.4 nm, 5.5 nm, 43.5 nm, and 95.9 nm, respectively. The Ra of the AlCrN coatings drops down from 58.7 nm to 37.8 nm as the bias voltage increases from 40 V to 80 V. Note that the surface roughness values of the AlCrN coatings prepared are lower than those of the AlCrN coatings in the references [29,30,31,32]. The Ra of the CrAlSiN was the lowest at 80 V of the substrate bias. It can be seen from Figure 7c that the coating surface had fewer peaks like bamboo shoots than that prepared by substrate biases Figure 7a,b,d, so the Ra of the CrAlSiN coating prepared by substrate bias of 80 V was the lowest. The protruding peaks in the three-dimensional surface morphology were the metal liquid droplets or particles on the coating surface, which was a typicalological characteristic of the coating deposited by the arc ion plating technology. Because of the high surface temperature of the target during the coating process, cathode arc spots appeared on the surface of the target, which made some metal liquid droplets or metal particles directly splash on the surface of the substrate [21]. The Ra of the coating increased with the increase in the substrate bias voltage. The number of metal particles escaping from the surface of the target increases sharply in unit time, and when there are too many metal particles, they can interact with N_2_ molecules in time and directly deposit on the surface of the substrate to form metal liquid droplets or particles. In addition, the enhancement of the substrate bias voltage will intensify the electric field and its repulsion effect, which prevents individual micro-particles from reaching the substrate due to the repulsion of the electric field. Simultaneously, the subsequent high-energy ion deposition leads to a decrease in the secondary sputtering of the early-deposited film which reduces the number of particles on the surface of the coating, causing some particles to fall off and forming needle-like pores.

**Figure 8:**
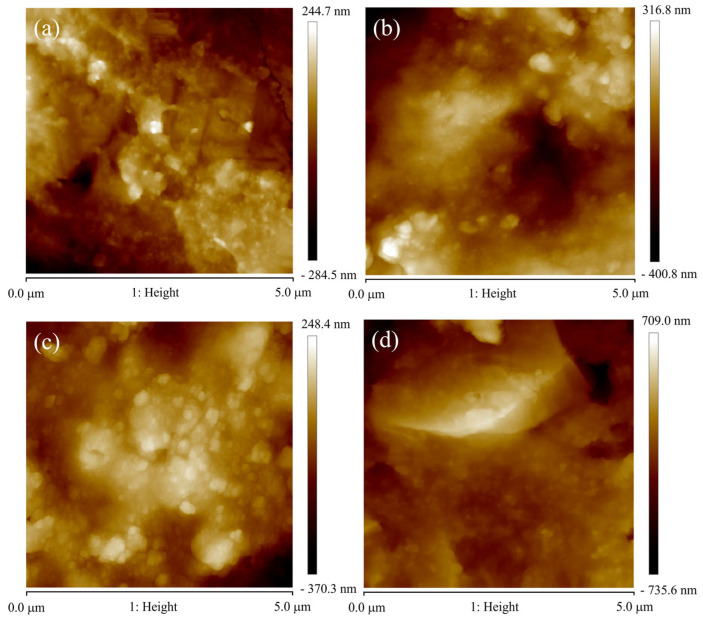
Two-dimensional surface topography of CrAlSiN composite coating with different substrate bias (**a**) 40 V, (**b**) 60 V, (**c**) 80 V, (**d**) 100 V.

Figure 8 shows the 2D surface morphology of CrAlSiN coatings prepared under different substrate biases. Based on the 2DAFM, the grain size is presented in Table 2. From Figure 8, it can be observed that the grains on the coating surface are distributed in an island-like aggregation state. Table 2 reveals that as the substrate bias voltage increases, the average grain size gradually increases from 117.2 nm to 219.4 nm, which is consistent with the trend observed in the previous XRD grain size results. However, the grain size is significantly larger than the results obtained from the Scherrer formula in XRD. The reason is that the agglomeration phenomenon occurs during grain growth. Additionally, the minimum grain size on the coating surface layer does not change significantly, but the maximum grain size increases significantly from 289.7 nm to 975 nm with the substrate bias voltage increasing. Therefore, as the substrate bias voltage increases, the tendency for grain agglomeration also increases. The reasons have been provided in the previous XRD and thickness analysis.

### 3.4. Sectional Morphology of the Coatings

**Figure 9:**
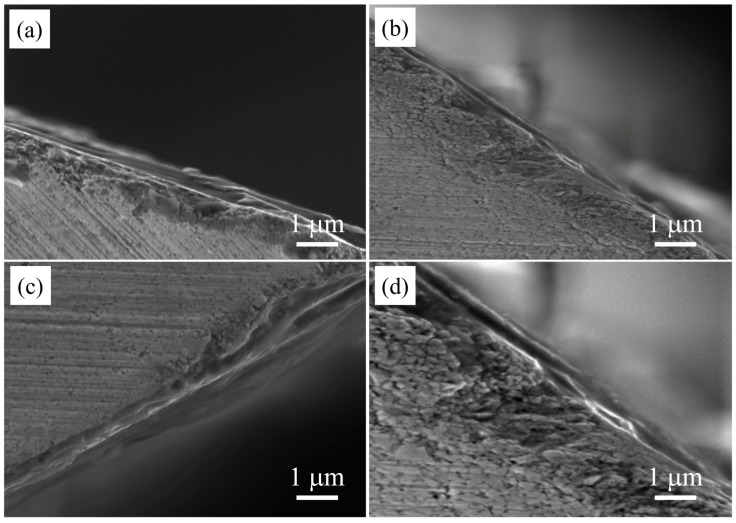
Section topography of CrAlSiN composite coating with different substrate biases: (**a**) 40 V, (**b**) 60 V, (**c**) 80 V, (**d**) 100 V.

Figure 9 shows the cross-sectional morphology of the coated cutting tool obtained by wire cutting. It can be seen from Figure 9 that the underlayer Cr was closely combined with the substrate, and the coating interface between each coating gradually became blurred with the increase in the substrate bias voltage. As can be seen from Figure 9c, the overall cross-sectional area of the coating prepared at 80 V of the substrate bias voltage was uniform and dense, with the smallest sample size and no large particles on the surface. The bonding-layer Cr was tightly combined with the substrate, which was related to the coating’s strong bonding force, fracture toughness, and resistance to plastic deformation. In Figure 9a, there were signs of partial peeling and detachment on the surface of the coating at 40 V of the substrate bias voltage. In Figure 9b, the peeling signs of coatings at 60 V disappeared, and the particles grew by a certain extent compared to those at 40 V. However, in Figure 9d, the coating particles at 100 V increased and reached the maximum value among the four kings of coatings, which was consistent with the previous AFM surface analysis.

With bias voltage increasing (from 40 V to 80 V), the mobility of the adatoms is enhanced, which makes some adatoms diffuse to the inter-grain voids to form denser and compacter coatings [33]. But during the deposition, the micro-particles likely absorb negative electrons, so the particles carry a large amount of negative charge before reaching the substrate. Therefore, as the bias voltage increases, the repulsive force between the negatively charged substrate and large particles increases [34], which is conducive to reducing the deposition of particles to form a large-particle loose coating shown in Figure 9d.

### 3.5. Coating Hardness

**Figure 10:**
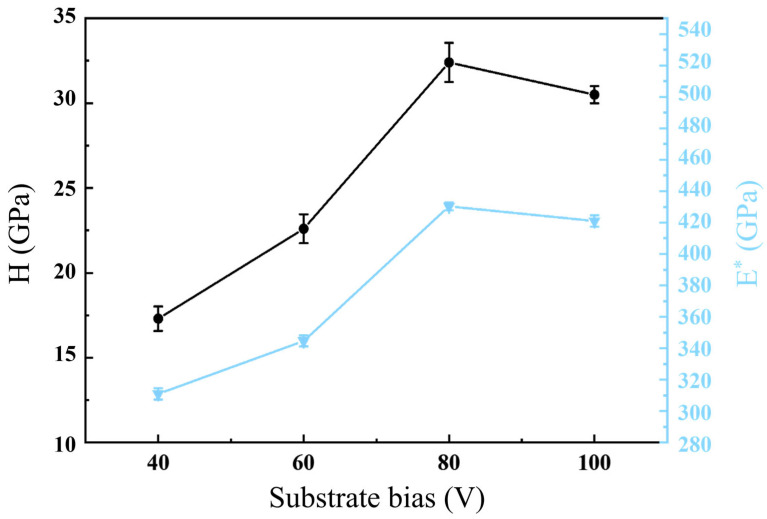
Nano-hardness and elastic modulus of CrAlSiN composite coatings with different substrate biases.

The nano-hardness and elastic modulus of the CrAlSiN composite coating were measured by nanoindentation method, and the results are shown in Figure 10 and Table 3. When the substrate bias increased from 40 V to 60 V, the coating hardness showed a slight upward trend, until the hardness reached the maximum value of about 31.30 GPa at 80 V. Phase structure, crystallite size, densification and nanocomposite structure all influence the hardness of coatings [35]. First, the replacement of Cr atoms by Al atoms and the solid-solution hardening effect are caused by grain refinement. Second, lattice distortion and stress increase the difficulty of dislocation extension. Third, the formation of nanocomposite structure contributes to enhancing hardness. This structure is composed of nanoparticles embedded in an amorphous matrix. Thus, it can be clearly seen from the data provided in Figure 3 that as silicon concentration increases, the amorphous fraction continuously rises. When the substrate bias was 100 V, the decrease in the hardness of the coating was directly attributed to the roughness of the coating, the formation of defects, and the sharp increase in the content of the amorphous phase. Y. Wang showed that Si_3_N_4_ has a low diffusion rate in the coating [19].

It was found that H/E* was in direct proportion to the fracture toughness of the coating, and H^3^/E*^2^ was in direct proportion the anti-plastic deformation of the coating [36,37]. According to the data in Table 3, the values of H/E and H^3^/E*^2^ for the four CrAlSiN coatings show the same trend as the hardness change with the change in substrate bias— they increase first and then decrease. This may be caused by the increasing substrate bias which improves the kinetic energy of argon ions, resulting in a stronger sputtering. And the surface atomic migration ability is improved, but the nucleation rate is reduced to ultimately form bigger grain structures, as shown in the XRD analysis. However, excessive bias voltage can lead to reverse sputtering and introduce defects, resulting in stress accumulation and decreased material plasticity. Overall, when the substrate bias is 80 V, the values of nano-hardness H, E*, H/E*, and H^3^/E*^2^ for the CrAlSiN composite coating are 31.30 GPa, 432.15 GPa, 0.0724 and 0.1642, respectively, which are a little higher than those prepared by magnetron sputtering [12] and HPPMS [38]. From the analyses in Figure 7 and Figure 8, we can see that CrAlSiN composite coating with substrate bias 80 V is denser and more compact and has fewer large grains, which is a benefit for the larger H/E* and H^3^/E*^2^. The smaller the grain size, the shorter the distance from the dislocation to the grain boundary, the smaller the number of dislocations in front of the dislocation, and the smaller the resistance to dislocation slip, thereby improving the ability to deform plastically. Moreover, nanocrystal dispersion, reduced microstructural defects, and increased thin film density collectively improve the mechanical properties [39,40].

### 3.6. Coating Adhesion

**Figure 11:**
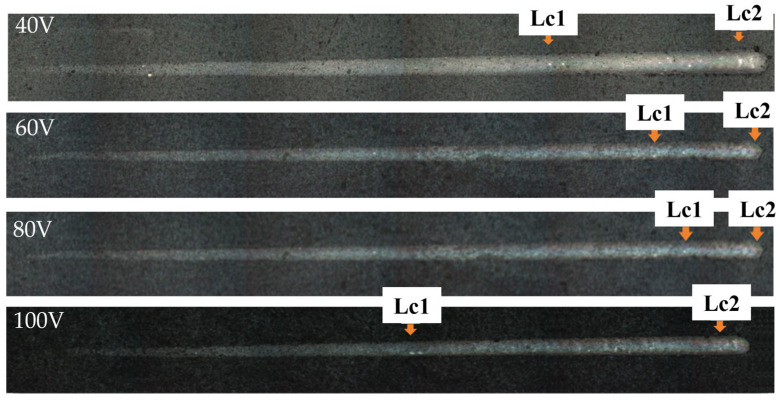
Nano-scratch morphology of CrAlSiN composite coatings with different substrate biases.

The size of the adhesion between substrates and coatings is the key to ensuring the normal service of the coatings. In this experiment, a nano-scratch tester was used to characterize the critical load. From the moment when the needle tip makes contact with the coating to the final unloading, the whole process is divided into three stages: the point of cohesive failure of the coating is denoted as Lc1; the point of adhesive failure of the coating is denoted as Lc2; and the point of contact between the punch and the substrate is denoted as Lc3. Lc2 indicates that the coating begins to appear off [41], which has great reference value, so Lc2 is used as the critical load of the coating in this experiment. The specific scratch morphology is shown in Figure 11.

**Figure 12:**
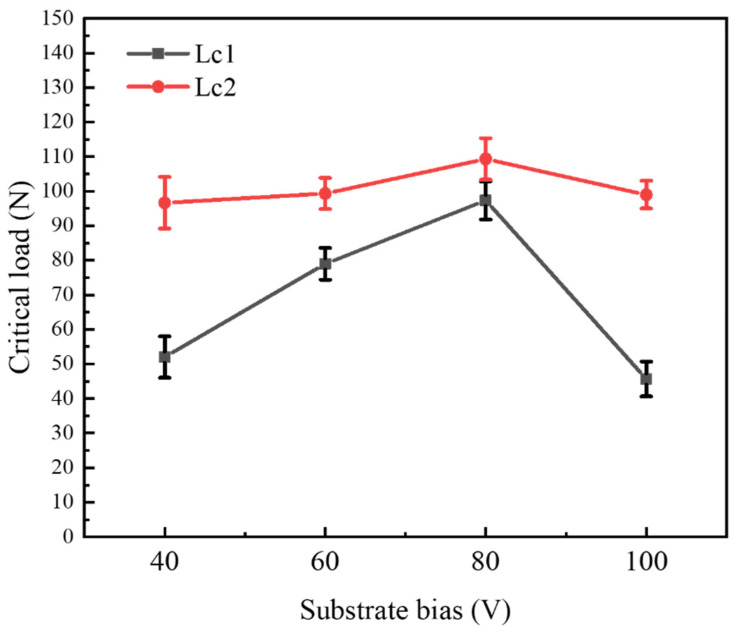
Variation trend of critical load of CrAlSiN composite coating with different substrate bias voltage.

Figure 12 shows the trend of critical load change in CrAlSiN composite coating with different substrate bias voltage during deposition. It can be seen that the critical load of the coating was greatly affected by the substrate bias voltage during deposition. As the substrate bias voltage increased to 80 V, the critical load of the coating increased from 96.14 N to 109.24 N. This is attributed to the increase in substrate bias voltage, which increases the energy obtained by the film-forming particles. The bombardment of high-energy particles is conducive to improving the bonding strength between the deposited particles and the substrate. High-energy particles can effectively peel the deposited particles with poor adhesion; the coating defects are reduced, and the structure is dense and compact. However, when the substrate bias voltage in the furnace rose to 100 V, the critical load of the coating decreased to 95.62 N. This is because there is a more obvious phenomenon of backscattering, and there are defects in the coating, resulting in a slight decrease in the critical load of the coating.

**Figure 13:**
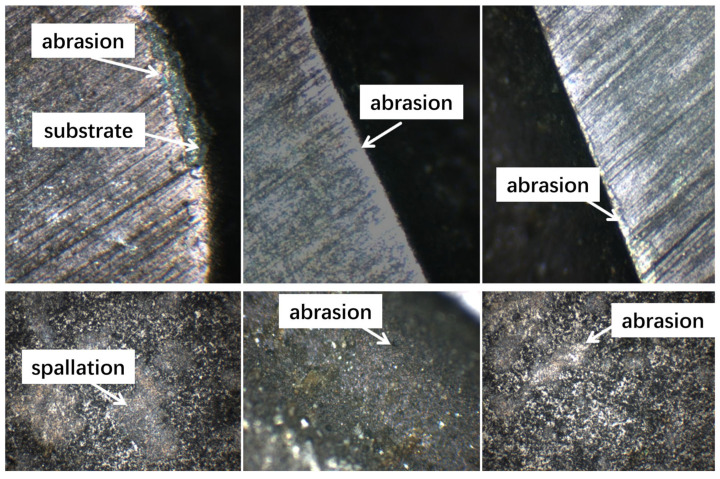
Wear status of side blade and bottom blade of three coated tools.

Figure 13 is the wear status of the side blade and bottom blade of three coated tools. After 60 min of cutting, the wear conditions of the three coated tools were different. Notably, the end mill with CrAlSiN coating at 60 V substrate bias voltage showed wear at the tip of the bottom edge, which could have been caused by multiple factors. First, dry cutting of brittle workpieces generates intense thermal shocks at the contact between the tool and the workpiece, causing the tool surface temperature to rise rapidly. This thermal shock will accelerate the peeling of the tool’s surface coating, making the tool more prone to wear or even breakage. Secondly, the bonding strength between the substrate and the costings at 60 V substrate bias voltage may be insufficient, possibly due to too low a substrate bias during the coating process. Therefore, during the cutting process, the hard particles generated from the workpiece continuously rub against the tool surface coating, further accelerating wear and spalling, which reduces the tool’s service life. In contrast, the coatings at 80 V substrate bias voltage, although showing wear, did not exhibit coating spalling. This may be caused by the substrate bias during the coating preparation, which was moderate, and the coating surface roughness was relatively low, maintaining the coating’s relative stability. The wear of the coating at 100 V substrate bias voltage was relatively slight, possibly because an excessive substrate bias during preparation caused a resputtering effect, increasing the coating surface roughness and thereby affecting the cutting performance.

**Figure 14:**
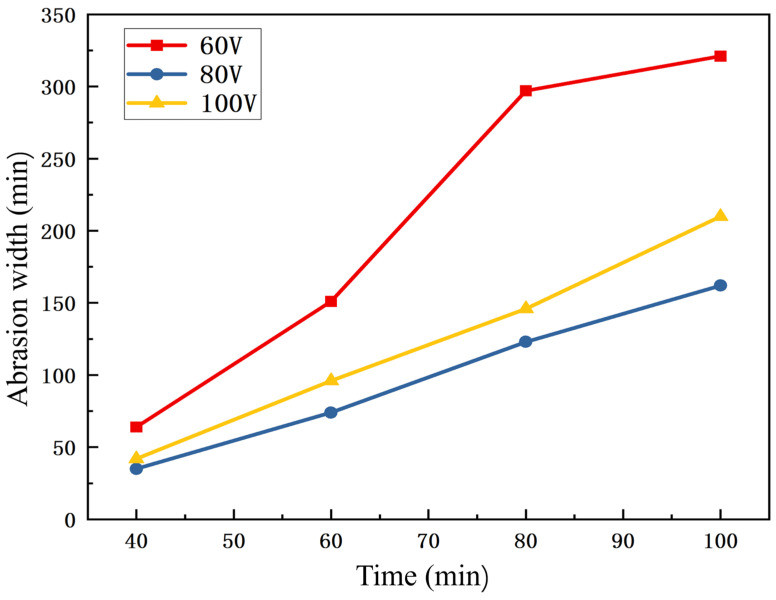
Side blade wear band width of the three coated vertical milling machines at different cutting times.

Microscopic observation, measurement and recording of the wear behavior of cutting tools during the cutting process enable a more accurate evaluation of tool service life and performance. According to the data in Figure 14, the width of the wear on the bottom cutting edge face was relatively small at a cutting time of 30 min, while the wear intensified gradually with the increase in cutting time. At a cutting time of 60 min, failure occurred on some coated tools; in particular, the tool prepared at 60 V substrate bias voltage even suffered from coating spallation, whereas the tool fabricated at 100 V substrate bias voltage exhibited significant wear. This phenomenon is mainly caused by the relatively high surface roughness of the tools, which induced large frictional force during cutting and thus accelerated the wear process. Notably, a comparison of the data in the Figure reveals that the coated tool prepared at 80 V substrate bias voltage possessed a relatively long service life. This could be ascribed to the more optimal preparation parameters adopted for the coatings at 80 V substrate bias voltage during the deposition process, which resulted in a lower surface roughness of the coating and thereby enhanced the wear resistance and service life of the tool.

**Figure 15:**
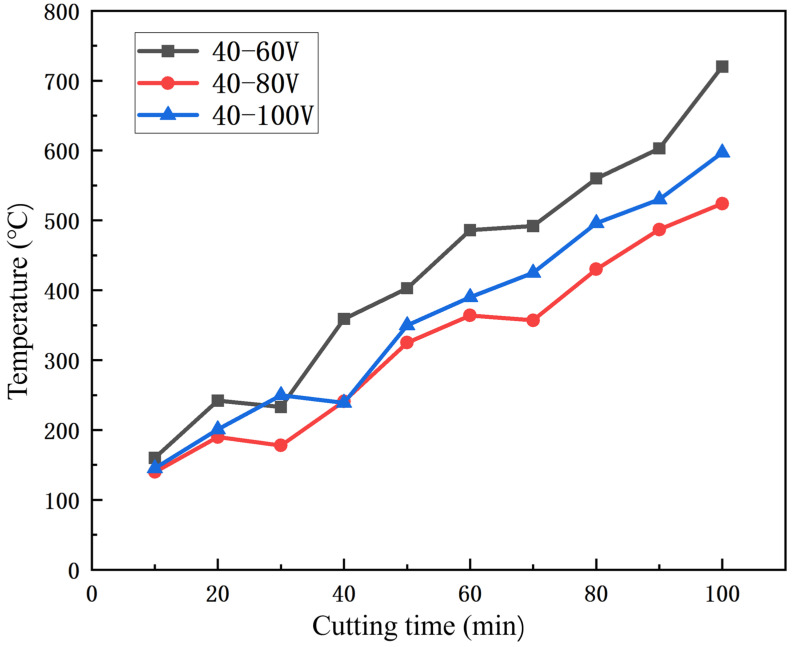
Cutting temperature of continuous cutting of 45 steel by coated milling cutter.

Figure 15 illustrates the variation in cutting temperature of the CrAlSiN nano-graded coated milling cutters with cutting time. Within the initial 30 min, the cutting temperatures of the three types of coated milling cutters exhibited negligible changes and increased gradually with the extension of cutting time. Notably, however, the cutting temperature of the coated milling cutter prepared at 60 V substrate bias voltage rose at a faster rate than that of the other two cutters, which was due to the onset of tool wear that induced an increase in frictional force between the cutter and 45 steel. After 40 min of cutting, the cutting temperature of the coated milling cutter fabricated at 100 V substrate bias voltage increased abruptly, with its rising rate being significantly higher than those of the other two cutters, indicating that the coated milling cutter at 100 V substrate bias voltage had entered the wear stage. When the cutting time reached 80 min, the milling cutter with CrAlSiN coatings at 60 V substrate bias voltage suffered from severe wear, which caused direct contact between the tool substrate and 45 steel and thus led to a sharp surge in cutting temperature. In contrast, the milling cutters with coatings at 80 V and 100 V substrate bias voltage had not yet met the criteria for complete failure.

## 4. Conclusions

CrAlSiN nanocomposite coatings were prepared by arc ion plating technique with four different substrate biases, and the effects of substrate on the microstructure and mechanical properties of the coatings were analyzed in detail; the following research results were obtained:

(1) The coating phase species of the four CrAlSiN nanocomposite coatings did not change with the change in substrate bias voltage and remained a single fcc-(Cr, Al) N phase. In addition, part of Al and Si were solid-dissolved in the CrN phase, and part of Si in the coating remained in the form of the amorphous phase. All the coatings showed a preferential orientation of the (200) crystal plane. It can be seen from cross-sectional morphology that the coating structure was the most dense at the substrate bias voltage of 80 V.
(2) With the increase in substrate bias, the hardness of the CrAlSiN coating showed an increasing and then decreasing trend. The initial increase in hardness can be attributed to the increase in substrate bias, which lead to a gradual decrease in grain size. However, the subsequent decrease in hardness may be due to the agglomeration of small particles inside the coating, forming larger hills, and the formation of defects such as pores in the coating.
(3) The variation trend of bonding force and hardness of the coating was the same. When the substrate bias was 80 V, the coating had the highest H (31.30 GPa), E* (432.15 GPa), H/E* (0.0724), H^3^/E*^2^ (0.1642), and bonding force (109.24 N), and the mechanical properties were the best.
(4) When the substrate bias was 80 V, the Lc1 and Lc2 in the coating’s critical load were all larger than those at 0 V, 60 V, and 100 V, and the bonding strength between the film and the substrate was relatively excellent.

## Figures and Tables

**Table 1:** Process parameters for CrAlSiN nanocomposite coatings with different substrate bias.

Process Parameters	Numerical Value
Distance between the substrate and target (mm)	300
Working pressure (Pa)	2.6
Bias voltage of the substrate (V)	40, 60, 80, 100
Deposition temperature (°C)	480
Revolution and rotation speed of the sample (r/min)	50
N_2_/Ar (sccm)	1100/260
Deposition time (min)	90

**Table 2:** The grain size of CrAlSiN nanocomposite coatings with different substrate bias.

Substrate Bias	Grain Size		
	Mean/nm	Minimum/nm	Maximum/nm
40 V	117.2	60.4	289.7
60 V	138.4	58.3	488.1
80 V	185.644	60.4	519.4
100 V	219.4	61.4	975

**Table 3:** Nano-hardness, elastic modulus, H/E*, H^3^/E*^2^ of CrAlSiN composite coatings with different substrate biases.

Matrix Bias (V)	H (GPa)	E* (GPa)	H/E*	H^3^/E*^2^
40 V	17.70	308.43	0.05734	0.0583
60 V	22.96	345.68	0.0664	0.1013
80 V	31.30	432.15	0.0724	0.1642
100 V	30.05	418.32	0.0718	0.1551

## Data Availability

The original contributions presented in this study are included in the article. Further inquiries can be directed to the corresponding author(s).
